# The Biogenic Synthesis of Bimetallic Ag/ZnO Nanoparticles: A Multifunctional Approach for Methyl Violet Photocatalytic Degradation and the Assessment of Antibacterial, Antioxidant, and Cytotoxicity Properties

**DOI:** 10.3390/nano13142079

**Published:** 2023-07-15

**Authors:** Muhammad Asjad Afzal, Muhammad Javed, Sadia Aroob, Tariq Javed, Maryam M. Alnoman, Walla Alelwani, Ismat Bibi, Muhammad Sharif, Muhammad Saleem, Muhammad Rizwan, Ahmad Raheel, Ihsan Maseeh, Sónia A. C. Carabineiro, Muhammad Babar Taj

**Affiliations:** 1 Institute of Chemistry, Green Synthesis Laboratory, The Islamia University of Bahawalpur, Bahawalpur 63100, Pakistan; asjadafzal7766@gmail.com (M.A.A.); sadiaaroob4991@gmail.com (S.A.); drismat@iub.edu.pk (I.B.); sharifqureshi34@gmail.com (M.S.); m.saleem@iub.edu.pk (M.S.); ihsanullah1742@gmail.com (I.M.); 2 Department of Chemistry, University of Lahore, Lahore 54590, Pakistan; javedqurashi1997@gmail.com (M.J.); muhammad.rizwan@chem.uil.esu.pk (M.R.); 3 Department of Chemistry, University of Sahiwal, Sahiwal 57000, Pakistan; mtariq@uosahiwal.edu.pk; 4 Department of Biology, Faculty of Science, Taibah University, Yanbu P.O. Box 344, Saudi Arabia; mnaaman@taibahu.edu.sa; 5 Department of Biochemistry, College of Science, University of Jeddah, Jeddah 21959, Saudi Arabia; welwani@uj.edu.sa; 6 Department of Chemistry, Quaid-e-Azam University, Islamabad 44000, Pakistan; ahmadraheel001@gmail.com; 7 LAQV-REQUIMTE, Department of Chemistry, NOVA School of Science and Technology, Universidade NOVA de Lisboa, 2829-516 Caparica, Portugal

**Keywords:** silver, zinc oxide, antibacterial, antioxidant, photocatalytic degradation, methyl violet

## Abstract

In this study, bimetallic nanoparticles (NPs) of silver (Ag) and zinc oxide (ZnO) were synthesized using *Leptadenia pyrotechnica* leaf extract for the first time. Monometallic NPs were also obtained for comparison. The characterization of the prepared NPs was carried out using various techniques, including UV-Visible spectroscopy (UV-Vis), scanning electron microscopy (SEM), and X-ray diffraction (XRD). The latter confirmed the crystalline nature and diameter of the monometallic and bimetallic NPs of Ag and ZnO. The SEM images of the prepared NPs revealed their different shapes. The biological activities of the NPs were evaluated concerning their antibacterial, antioxidant, and cytotoxic properties. The antibacterial activities were measured using the time-killing method. The results demonstrated that both the monometallic and bimetallic NPs inhibited the growth of Gram-negative (*Escherichia coli*) and Gram-positive (*Staphylococcus aureus*) bacteria. The antioxidant activities of the NPs were evaluated using the DPPH (2,2-diphenyl-1-picrylhydrazyl) assay and their cytotoxicity was checked using the MTT (3-(4,5-dimethylthiazol-2-yl)-2,5-diphenyltetrazolium bromide) assay. The results indicated that the controlled quantity of the monometallic and bimetallic NPs did not affect the viability of the cells. However, the decreased cell (L-929) viability suggested that the NPs could have anticancer properties. Furthermore, the photocatalytic degradation of methyl violet and 4-nitrophenol was investigated using the prepared Ag/ZnO NPs, examining the factors affecting the degradation process and conducting a kinetic and thermodynamic study. The prepared Ag/ZnO NPs demonstrated good photocatalytic degradation (88.9%) of the methyl violet (rate constant of 0.0183 min^−1^) in comparison to 4-nitrophenol (NPh), with a degradation rate of 81.37% and 0.0172 min^−1^, respectively. Overall, the bimetallic NPs showed superior antibacterial, antioxidant, cytotoxic, and photocatalytic properties compared to the monometallic NPs of Ag and ZnO.

## 1. Introduction

Researchers are currently faced with a significant hurdle in their work, which revolves around the growing apprehension regarding the toxicity of metallic nanoparticles generated through physical and chemical methods [1,2]. However, there is a promising avenue that offers both environmental benefits and enhanced safety—the green production of nanoparticles. By shifting away from the utilization of hazardous chemicals, scientists are exploring the utilization of reducing agents derived from plant sources to synthesize nanoparticles [3,4]. This innovative approach not only addresses concerns about toxicity, but also aligns with the principles of sustainability and eco-friendliness. Nanotechnology, specifically linked to “Green Synthesis”, has significantly advanced the field of nanoscience, leading to remarkable improvements in diverse areas such as environmental, medicinal, and pharmacological chemistry. The unique chemical and physical properties of nanomaterials have garnered attention due to their size, morphology, and geometry, clearly distinguishing them from bulk materials. The synthesis of nanoparticles (NPs) has produced numerous useful applications in catalysis, waste management, environmental contamination, and the control of human infections. Catalytic materials are becoming increasingly important due to the growing demand for resource conservation, energy production, and environmental cleansing [5,6,7,8,9,10,11,12].

As the world’s population continues to grow and industrial development keeps expanding, environmental pollution is becoming a significant issue. Many hazardous organic pollutants enter the environment through various industries, including organic dyes. These pollutants are a significant environmental concern due to their high toxicity, carcinogenicity, durability, and low biodegradability, with their removal from the environment being a priority.

Some examples are dyes and organic compounds. One example of an organic compound is 4-nitrophenol (NPh) [13]. It is often used as an intermediate in the synthesis of various organic compounds, such as drugs and insecticides, or as a reagent in laboratory experiments [14]. It can cause adverse effects on various organs, including the liver, kidneys, and central nervous system, and also skin, eye, and respiratory tract irritation [15]. NPh is also harmful to aquatic organisms and can have negative impacts on ecosystems if it is released into water bodies [16].

Methyl violet is an example of a cationic dye. It has a dark green color in powdered form. MV has found its use in various industries such as textiles, printing, leather, and rubber, etc. MV is a recognized irritant that can injure the eyes, skin, gastrointestinal, and respiratory systems, in addition to being mutagenic and carcinogenic [17,18]. Additionally, MV prevents the growth of microorganisms and negatively influences the photosynthetic processes in aquatic environment [19]. Various treatment methods for the degradation of dyes have been reported.

Previous research has shown that photocatalytic degradation under sunlight is an effective method for the degradation of organic pollutants to non-toxic substances. Photocatalytic degradation is cost-effective and does not require complex instrumentation. The green synthesis of metal NPs is more practical and economic, due to their biocompatibility and catalytic behavior [20,21,22,23,24,25,26].

Among the metal nanoparticles, Ag and ZnO NPs are receiving higher attention due to their wide range of applications, such as personal care products (PCP), food storage containers, wound dressing, water purification, drug delivery, bio-sensing, and textiles, etc. Noble metal NPs, such as Ag NPs, are well recognized as redox catalysts, involving less activation energy and offering a distinct path for the electron transfer reaction. The high surface energy provided by Ag NPs supports surface reactivity. The green synthesis of Ag NPs using *Trigonella foenum-graecum* seed extract was reported by Awad et al. [27], who described their effects on the photodegradation of Rhodamine B dye. The results showed that nearly 93% of the Rhodamine B was degraded after 216 h.

On the other hand, increased bacterial infections, the drug resistance of bacteria, and intolerable toxicity brought about by an enforcement of the high-dose administration of antibiotics have led researchers to approach nanotechnology-based therapeutics. Among all the metallic NPs, the most important are NPs of Ag [8,9,27,28], ZnO [2,29], and Cu [30]. In particular, Ag and ZnO NPs are very effective against pathogens when used in small quantities. Due to their environmentally friendly nature and non-toxic preparation procedures, these Ag and ZnO NPs are the best alternatives for several biological uses. Both show antibacterial, anti-fungal, and anti-cancerous properties. Thus, both Ag and ZnO NPs are important for mankind’s well-being, which contributed to the choice to use them in our study. The synthesis of Ag/ZnO bimetallic NPs has been performed in many physical and chemical ways, such as reduction via the solvothermal method, hydrazine hydrate, and ultrasonic irradiation, but biosynthetic methods are still missing [28,31,32,33,34].

This study deals with the unprecedented green synthesis and characterization of bimetallic NPs of Ag and ZnO using *Leptadenia pyrotechnica* (*L. pyrotechnica*) plant extract to reduce silver nitrate and zinc acetate dehydrate and form NPs. Thus, a green strategy was used to prepare environmentally friendly and cost-effective NPs, with advantages over the other methods used for the preparation of this type of materials. *L. pyrotechnica* is rich in phytochemicals, such as flavonoids, phenolic acids, cardiac glycosides, pregnane glycosides, alkaloids, fatty acids, terpenes, sterols, hydrocarbons, amino acids, and sugars, which help in the reduction and stabilization of metals [35]. This plant also has several medicinal uses [36,37]

The synthesis of the NPs was performed using this plant for the first time and the effectiveness of the prepared materials was tested using biological assays and the photocatalytic degradation of methyl violet (MV) and 4-nitrophenol (NPh), which were used as models of dyes and organic compounds, respectively. The study offers a fresh way of investigating the applications and combinatorial effects of Ag and Zn in nanoparticle configurations.

## 2. Materials and Methods

### 2.1. Materials

High-purity chemicals were used in this study: zinc acetate dehydrate, silver nitrate, sodium hydroxide, zinc chloride, and liquid ammonia with a 99.99% purity were obtained from Sigma Aldrich and used without further purification.

### 2.2. Preparation of Leaf Extract of L. Pyrotechnica

*L. pyrotechnica* plants were freshly collected from the local area of the Islamia University of Bahawalpur, Pakistan, and authenticated by the Botany Department of the University. To prepare the leaf extract, the leaves were first cleaned with distilled water to remove any dust and then dried for 24 h. After drying, the leaves were soaked in distilled water and heated at 40  ±  5  °C for 2 h with stirring. The resulting mixture was then filtered (with filter paper) to obtain the leaf extract. This extract was then used to prepare the monometallic (MNPs) and bimetallic (BNPs) nanoparticles.

### 2.3. Preparation of Monometallic NPs

**Figure 1:**
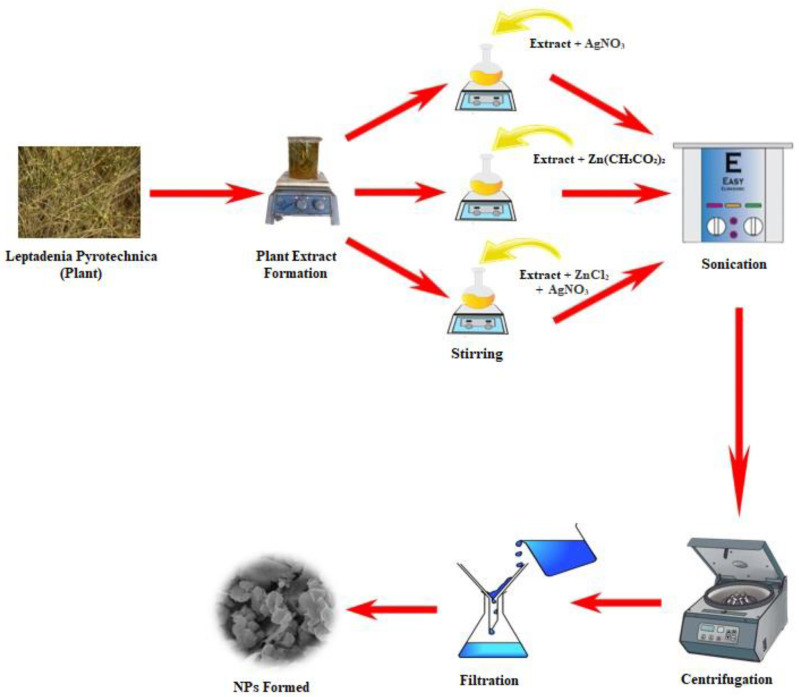
Synthesis of Ag NPs and Ag/ZnO NPs.

A solution of AgNO_3_ was prepared and added to 50 mL of *L. pyrotechnica* leaf extract that was heated on a hot plate with regular stirring. The temperature of the heating extract was raised to 60  ±  5  °C and the reaction was carried out for ~2 h with constant stirring. During the reaction, the extract reduced the salts, resulting in the formation of monometallic Ag NPs (Figure 1). The same procedure was used for the synthesis of the monometallic ZnO nanoparticles. A solution of 0.1 M zinc acetate was added to 50 mL of extract and 0.1 M NaOH was added to adjust the pH to 11. The reaction mixture was heated for 2 h with constant stirring, resulting in the formation of ZnO NPs. Both solutions were centrifuged for 12 min, washed with distilled water, and dried.

### 2.4. Preparation of Bimetallic NPs

In order to prepare the Ag/ZnO bimetallic nanoparticles (Figure 1), a 0.2 M solution of zinc chloride and 0.001 M solution of silver nitrate were used. The synthesis of the bimetallic NPs was performed by first heating 50 mL of the *L. pyrotechnica* leaf extract on a hot plate with constant stirring until it reached 60  ±  5 °C. Then, 50 mL of the ZnCl_2_ solution was added to the extract and allowed to react for 5 min. Then, 50 mL of the silver nitrate solution was added to the mixture and the reaction was carried out for 2 h with constant stirring. The resulting bimetallic NPs were then centrifuged for 12 min at 13,000 rpm and washed twice with distilled water. Finally, the particles were dried in an oven and ground for use in biological assays, the photocatalytic degradation of MV, and characterization.

### 2.5. Characterization of MNPs and BNPs

A spectroscopic analysis of the monometallic and bimetallic nanoparticles was conducted using a UV-Vis spectrophotometer (Apel PD303 UV) that operated within the range of 200–800 nm. An X-ray diffractometer (Bruker D8 Advance PXRD), with a Cu-Kα radiation source and wavelength λ equal to 1.540598 Å, was used for the XRD analysis of the nanoparticles. The average particle size was calculated using the Scherrer equation:

$$D=\frac{k\;\lambda \;}{\beta \;\cos\;\theta }$$

where *D* is the average size of the crystalline particles; *k* is a dimensionless shape factor close to 1, which depends on the shape of the particles (typically assumed to be around 0.9, but varying with the shape of the crystallite); *λ* is the wavelength of the X-rays used in the experiment; *β* is the full width at half maximum (FWHM) of the diffraction peak, in radians; and *θ* is the Bragg angle (between the incident X-ray beam and crystal lattice planes).

For the SEM analysis, the nanoparticles were first centrifuged at 10,000 rpm and then mixed in either methanol or water to obtain pellets. The pellets were then layered on a copper grid coated with carbon and dried before analysis.

### 2.6. Antibacterial Activity

The antimicrobial activity of the synthesized nanoparticles, against both Gram-negative and Gram-positive bacteria, was evaluated using a time-killing assay [38,39,40]. *Escherichia coli (E. coli)*, a Gram-negative bacterium, and *Staphylococcus aureus (S. aureus)*, a Gram-positive bacterium, were aerobically grown in liquid media at 37 °C. A solution of the synthesized nanoparticles in distilled water (200 μg/mL) was prepared and exposed to the bacterial cultures. The growth of the bacteria was assessed by measuring the absorbance at 600 nm using a spectrophotometer. A control sample without nanoparticles was also included in the assay.

### 2.7. Antioxidant Activity

To assess the scavenging effect of each sample, a 2,2-diphenyl-1-picrylhydrazyl (DPPH) assay was conducted with a slight modification. Samples in different concentrations (ranging from 10 to 100 μg/mL) and butylated hydroxytoluene (BHT) were dissolved in separate test tubes. Then, a solution of DPPH in ethanol (0.1 mmol/L) was added to each tube and thoroughly mixed. The tubes were then incubated in a dark place for ~30 min. The absorbance of each sample was measured at 517 nm using a spectrophotometer. The percentage of the inhibition effect was calculated using the following formula:

(1) $$DPPP\;inhibition\;\left(\%\right)=\frac{OD_{control}-OD_{sample}}{OD_{control}}\times 100\;\;$$

where *OD_control_* is the optical density (*OD*) of the control (*DPPH* solution in ethanol without any sample) and *OD_sample_* is the optical density of the sample being tested.

### 2.8. Cytotoxicity

The viability of the fibroblast normal cell line (L-929) was assessed using the prepared samples. L-929 cells were cultured in flasks containing M-199 (cell culture media) with 10% fetal bovine serum (FBS) and DMEM (Dulbecco’s Modified Eagle’s Medium) and incubated overnight with atmospheric CO_2_ (5%), at 37 °C. After this incubation, trypsin was added to the cells to separate them for 2–3 min, followed by centrifugation at 80 rpm for 10 min. The number of separated cells was estimated and 5000 cells were applied to 96-well plates (ELISA) and incubated for 24 h. The samples were tested for their toxicity in various concentrations ranging from 100 to 700 (mg/mL) by measuring the adenosine triphosphate (ATP) of the cells, the mitochondrial damage, and the reactive oxygen species (ROS) increment (dose-dependent). The cell viabilities were checked by adding MTT solution at a concentration of 200 (ml/L) and incubation for 3–4 h. DMSO (200 mL) was added to the MTT solution and the product was kept in the dark for 15–20 min. The OD of the post-incubation product was recorded at 595 nm.

### 2.9. Photocatalytic Activity

To investigate the photocatalytic activity of the prepared Ag/ZnO nanoparticles, their ability to degrade an organic agent under natural solar light (with an average flux of 500 W/m^2^) was tested. MV was used for the destabilization test. In total, 5 mg of the nanomaterial was mixed with a 10 ppm solution of the dyes and the resulting suspension was stirred in the dark for 10 min, before using sunlight, in order to establish an adsorption–desorption equilibrium. Afterwards, 2 mL of the mixture was withdrawn and its absorption peak was measured using a UV-visible spectrophotometer. This process was repeated at 10 min intervals, by removing 2 mL of the solution and measuring the absorption spectra. Then, the samples were placed in sunlight. After 120 min of reaction under sunlight, the experiment was stopped to determine the maximum degradation. The total percentage removal of the dye was calculated using the following formula:

$$Degradation\;\left(\%\right)=\frac{A_{0}-A_{t}}{A_{0}}\times 100$$

where *A*_0_ is the absorbance at time zero and *A_t_* is the absorbance at time *t*.

The comparison experiments performed for NPh were carried out in a similar way.

## 3. Results and Discussion

### 3.1. UV-Vis Monometallic and Bimetallic Nanoparticles

**Figure 2:**
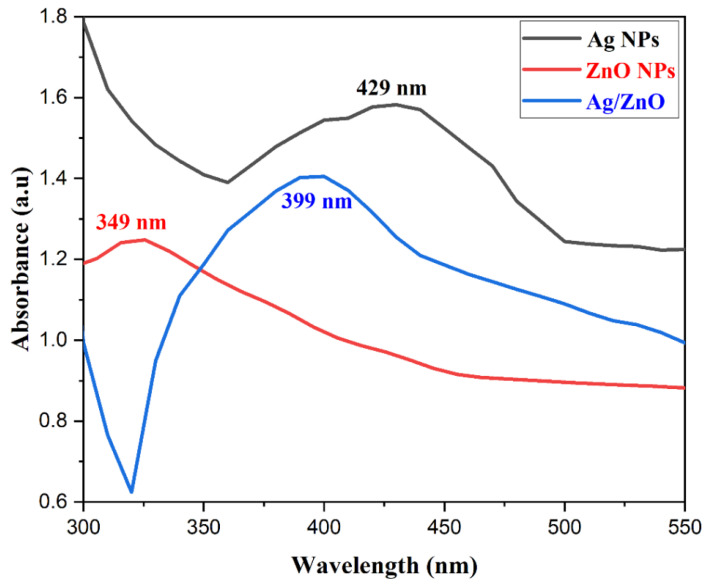
UV-Vis spectra of Ag, ZnO, and Ag/ZnO nanoparticles.

The samples were analyzed using ultraviolet-visible (UV-Vis) spectroscopy and the results are shown in Figure 2. The spectroscopic analysis of the synthesized Ag NPs was obtained in the wavelength range of 360–510 nm, with the strongest absorption peak appearing at 429 nm due to the surface plasmon resonance (SPR) phenomenon. This was due to the oscillation of the free electrons in the Ag NPs [41].

The UV-Vis spectrum of the ZnO NPs was obtained in the wavelength range of 330–480 nm, with the peak absorption being observed at 349 nm. The shift towards a lower wavelength indicated a reduction in the size of the ZnO NPs compared to the bulk ZnO [29].

Moreover, the UV-Vis spectrum of the Ag/ZnO NPs was recorded in the wavelength range of 300–700 nm, exhibiting an intense absorption peak at 399 nm, which shows the successful synthesis of the Ag/ZnO NPs.

### 3.2. XRD of Monometallic and Bimetallic Nanoparticles

**Figure 3:**
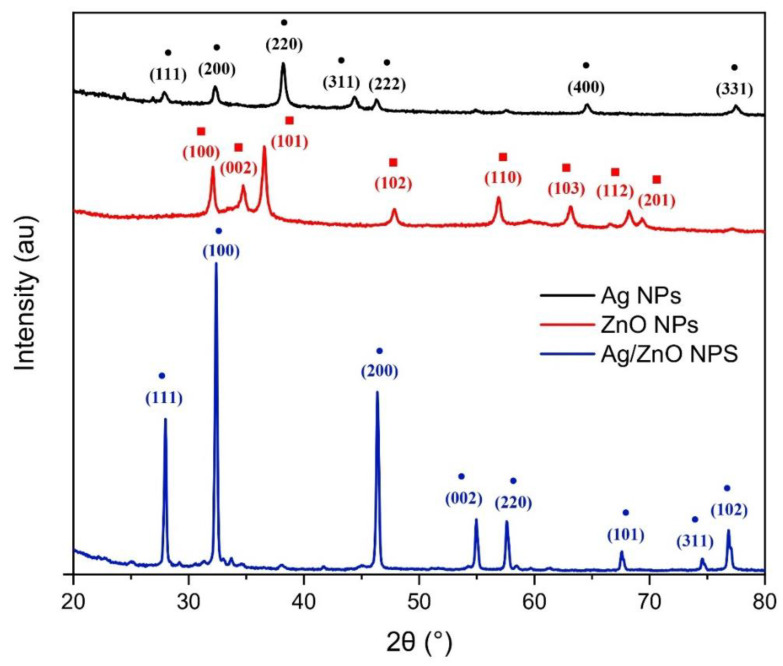
XRD spectra of monometallic and bimetallic nanoparticles. Squares are indicative of the Ag (FCC structure), while circles are of ZnO (hexagonal wurtzite structure).

The XRD diffractograms of the synthesized Ag NPs are displayed in Figure 3. The pattern confirms the crystalline and metallic nature of the silver monometallic nanoparticles. The XRD pattern of the Ag NPs displays (111), (200), (220), (311), (222), (400), and (331) lattice planes at different 2θ values of 27.87°, 32.29°, 38.31°, 44.49°, 46.26° 64.61°, and 77.53°. The peak at (111) is more pronounced than the peaks at (200) and (220), indicating an FCC (face-centered cubic) structure [42].

As is also shown in Figure 3, the ZnO NPs reveal several lattice planes of (100), (002), (101), (102), (110), (103), (112), and (201) at different 2θ values of 31.83°, 34.42°, 36.27°, 47.48°, 56.52°, 62.70°, 66.81°, and 69.32°. All the sample peaks correspond to the distinctive structure of the hexagonal wurtzite ZnO nanoparticles. According to the Scherrer equation, the estimated average particle size of the ZnO nanoparticles is 21.9 nm [43].

The XRD pattern of the bimetallic nanoparticles of zinc oxide and silver is also presented in Figure 3. The diffractogram displays different lattice planes of (111), (100), (200), (002), (220), (101), (311), and (102) at different 2θ values of 27.97°, 31.83°, 44.49°, 54.95°, 56.52°, 66.81°, 74.60°, and 77.53°. The (100), (110), and (112) crystalline planes indicate the hexagonal wurtzite structure of the ZnO nanoparticles, while the (200) and (311) planes show the FCC structure due to the silver NPs. The average particle size of the ZnO nanoparticles was estimated to be 196.3 nm.

### 3.3. SEM of Bimetallic Nanoparticles

**Figure 4:**
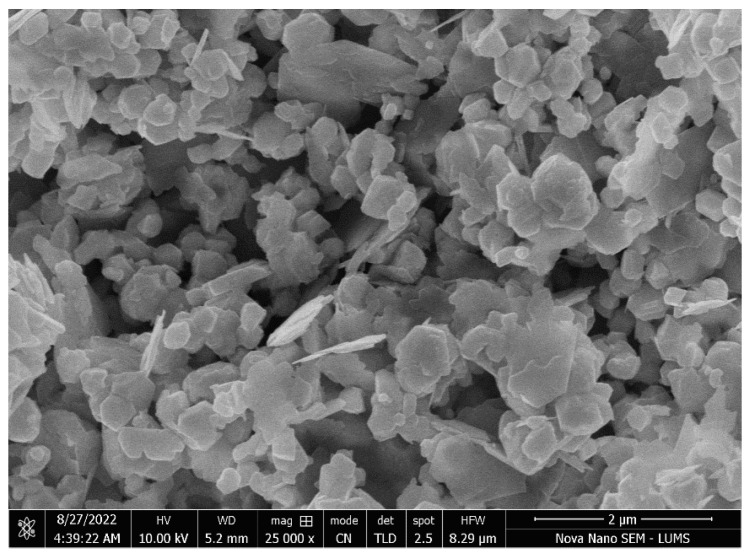
SEM image of Ag/ZnO NPs.

SEM was performed to investigate the morphology of the Ag/ZnO bimetallic nanoparticles. A representative image is presented in Figure 4, showing mostly spherical and well-dispersed particles with an average size of 397.5 nm. The observed particle size range is suitable for various advanced technological applications. Thus, Ag/ZnO bimetallic nanoparticles show desirable physical properties that make them potentially useful for various applications.

### 3.4. Time-Killing Assay (Antibacterial Activity)

**Figure 5:**
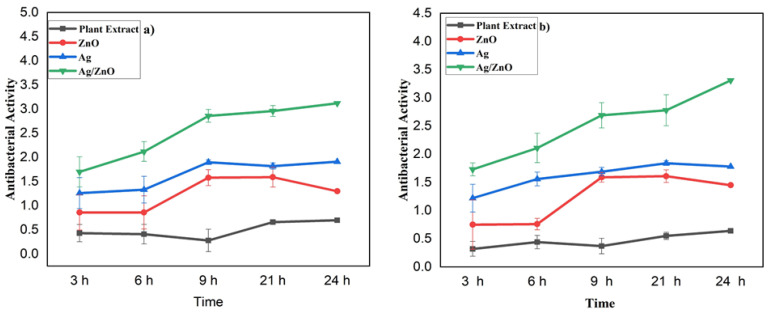
Antibacterial activity (against *S. aureus* and *E. coli*) of nanoparticles and plant extract at different incubation periods. The reported values were derived from triplicate samples. To accurately represent the data, the mean values are presented, along with their corresponding standard deviation, indicated as error bars.

A time-killing assay was used to evaluate the antibacterial activity of the monometallic and bimetallic nanoparticles. The results are summarized in Figure 5. All the nanoparticles demonstrated antibacterial activity against the Gram-positive bacteria *S. aureus*, with the bimetallic Ag/ZnO nanoparticles showing the strongest activity. Specifically, at a concentration of 1.70 µg/mL after 3 h of incubation, the Ag/ZnO NPs killed more bacteria compared to the other samples, including the plant extract, monometallic Ag NPs, and monometallic ZnO NPs. The plant extract killed bacteria at a concentration of 0.43 µg/mL, the monometallic ZnO NPs at 0.86 µg/mL, and the monometallic Ag NPs at 1.26 µg/mL. After 6 h of incubation, the plant extract killed bacteria at a concentration of 0.41 µg/mL, the ZnO NPs at 0.86 µg/mL, the Ag NPs at 1.33 µg/mL, and the BNPs at 2.12 µg/mL. After 9 h, the plant extract killed bacteria at 0.28 µg/mL, the ZnO NPs at 1.58 µg/mL, the Ag NPs at 1.90 µg/mL, and the BNPs at 2.86 µg/mL. After 21 h, the BNPs killed bacteria at a concentration of 2.96 µg/mL. The decreasing order of antibacterial power against *S. aureus* for all the samples, after 3, 6, 9, 21, and 24 h of incubation, is: Ag/ZnO NPs > Ag NPs > ZnO NPs > plant extract.

Overall, the results indicate that the bimetallic nanoparticles exhibited the strongest antibacterial activity against *S. aureus*. The monometallic Ag NPs also showed strong antibacterial activity, whereas the plant extract exhibited the least antibacterial activity.

Figure 5b shows the results of testing the MNPs and BNPs, prepared from *L. pyrotechnica* leaf extract, against the Gram-negative bacteria *E. coli*, in order to evaluate their antibacterial activities. The BNPs exhibited strong antibacterial activity against *E. coli*, similar to their activity against *S. aureus*. The antibacterial activity of the plant extract was the weakest among all the samples, as indicated in Figure 5.

After 3 h of incubation, the BNPs killed *S. aureus* at a concentration of 1.73 µg/mL, while the plant extract killed at a concentration of 0.32 µg/mL, the ZnO NPs killed at 0.75 µg/mL, and the Ag NPs killed at 1.22 µg/mL. After 24 h of incubation, the MNPs and BNPs killed bacteria at concentrations of 3.31 µg/mL, 1.78 µg/mL, and 1.45 µg/mL, respectively. The plant extract killed bacteria at a concentration of 0.64 µg/mL after 24 h of incubation. The antibacterial activity of the MNPs and BNPs, as well as the plant extract, after 6, 9, and 12 h of incubation, is presented in Figure 5.

The order of decreasing antibacterial power (against *E. coli*), after 3, 6, 9, 21, and 24 h of incubation, is: Ag/ZnO NPs > Ag NPs > ZnO NPs > plant extract

Overall, the results demonstrate that the bimetallic nanoparticles, particularly the Ag/ZnO NPs, had high antibacterial activity against both Gram-positive and Gram-negative bacteria. In opposition, the plant extract exhibited the weakest antibacterial activity among all the samples.

### 3.5. DPPH Radical Scavenging Activity Assay (Antioxidant Activity)

**Figure 6:**
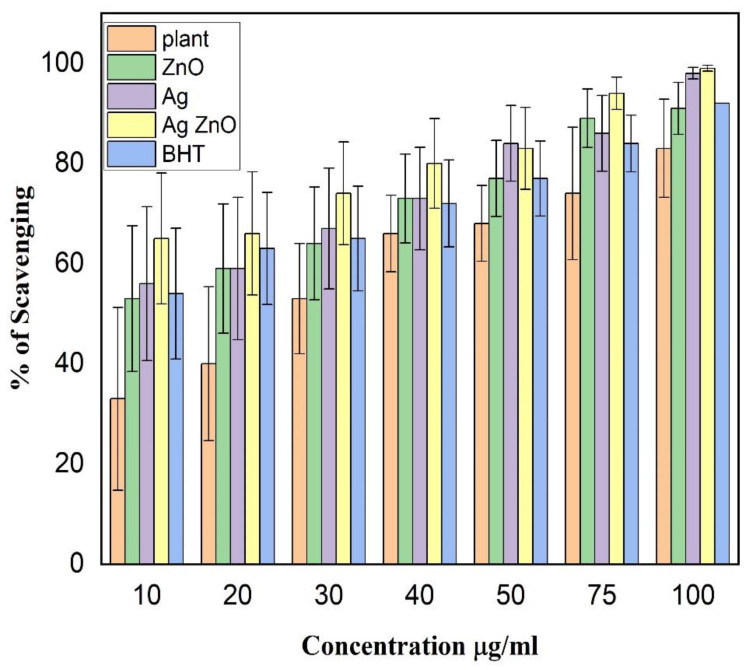
Variation of the scavenging effect (%) with the concentration for different samples. The reported values were derived from triplicate samples. To accurately represent the data, the mean values are presented, along with their corresponding standard deviation, indicated as error bars.

In order to evaluate the antioxidant activity of the monometallic and bimetallic NPs prepared from the leaf extract of *L. pyrotechnica*, different concentrations of the samples were tested using DPPH. The results showed that the BNPs of Ag and ZnO exhibited the highest scavenging effect of 99% at a concentration of 100 µg/mL, while the lowest scavenging effect of 83% was observed for the plant extract at the same concentration (Figure 6). At a concentration of 100 µg/mL, the scavenging effect of the ZnO NPs was 91%, the Ag NPs was 98%, and the BHT (standard) was 92%.

The antioxidant activity of the bimetallic NPs was also high compared to the monometallic NPs of ZnO and Ag and the standard BHT, at a low concentration of 10 µg/mL. At this concentration, the scavenging effect of the Ag/ZnO NPs was the highest (65%), followed by the Ag NPs (56%), the BHT (54%), the ZnO NPs (53%), and the plant extract (33%). The percentage of the scavenging effect varied at different concentrations of samples (Figure 6). The presence of different phytochemicals in the samples may account for the differences in their antioxidant scavenging effect.

In summary, the order of the decreasing scavenging effect at low and high concentrations of the samples is:

A low concentration (10 µg/mL): Ag/ZnO NPs (65%) > Ag NPs (56%) > BHT (54%) > ZnO NPs (53%) > plant extract (33%)

A high concentration (100 µg/mL): Ag/ZnO NPs (99%) > Ag NPs (98%) > BHT (92%) > ZnO NPs (91%) > plant extract (83%)

### 3.6. MTT Assay for Cytotoxicity

**Figure 7:**
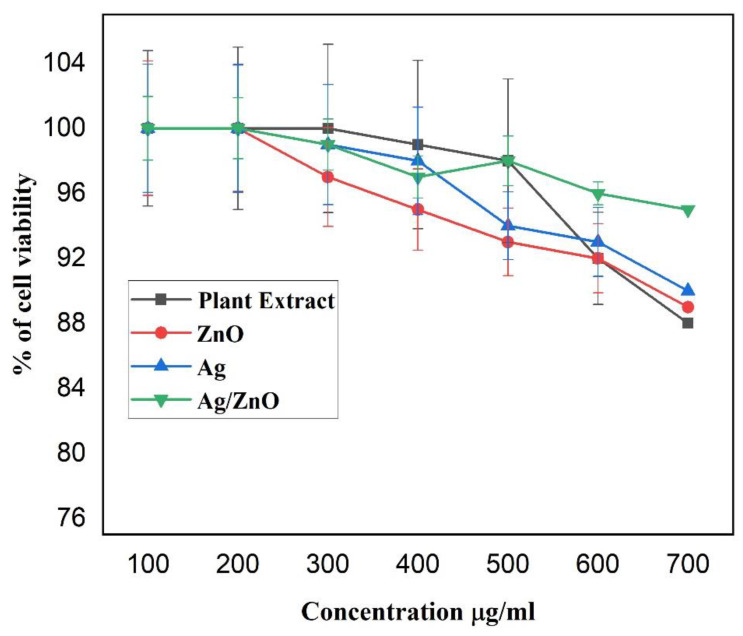
Variation of concentration of samples and % of cell viability. The reported values were derived from triplicate samples. To accurately represent the data, the mean values are presented, along with their corresponding standard deviation, indicated as error bars.

The cytotoxic effect of the monometallic and bimetallic nanoparticles, prepared from the leaf extract of *L. pyrotechnica*, was assessed using the MTT solution at various concentrations (Figure 7). The viability of the normal fibroblast cell lines (L-929) was determined using different concentrations of samples, ranging from 100 to 700 µg/mL. The results revealed that the cell viability decreased with an increase in the sample concentration. The viability remained at 100% when the plant extract, Ag MNPs, ZnO MNPs, and Ag/ZnO BNPs were used at 100 and 200 µg/mL. However, the cell viability decreased to 99%, 98%, 92%, and 88% with increasing concentrations of plant extract (400 to 700 µg/mL).

The plant extract did not show any harmful effects on the cells at concentrations of 100, 200, and 300 µg/mL. However, the plant extract above 300 µg/mL reduced the cell viability. The viabilities of the cells treated with the ZnO NPs were 100%, 100%, 97%, 95%, 93%, 92%, and 89%, at concentrations ranging from 100 to 700 µg/mL. For the Ag NPs, at concentrations ranging from 100 to 700 µg/mL, the cell viabilities were 100%, 100%, 99%, 98%, 94%, 93%, and 90%, for the same concentrations. The Ag/ZnO NPs showed viabilities in the range of 100%, 100%, 99%, 97%, 98%, 96%, and 95% in the same range.

Interestingly, the BNPs showed a higher percentage of cell viability than the plant extract and monometallic NPs of silver and zinc oxide, even at higher concentrations. In particular, using a concentration of 700 µg/mL, only the bimetallic NPs showed a high percentage of viability (95%), while the plant extract exhibited a lower percentage of viability (88%). The monometallic nanoparticles showed an intermediate percentage of cell viability between the plant extract and bimetallic nanoparticles. These findings suggest that the MNPs and BNPs showed anticancer properties, as indicated by their decreased cell (L-929) viability.

### 3.7. Photocatalytic Degradation of MV and NPh

**Figure 8:**
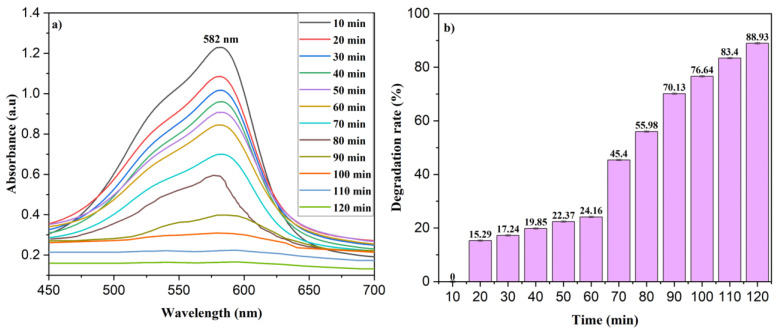
(**a**) Photocatalytic degradation and (**b**) degradation rate (%) of MV dye on Ag/ZnO NPs. The reported values on the left were derived from triplicate samples. To accurately represent the data, the mean values are presented, along with their corresponding standard deviation, indicated as error bars.

The ability of the Ag/ZnO NPs to degrade MV was investigated by adding 5 mg of the nanoparticles to destabilize 10 ppm of the MV within 120 min. The degradation rate was monitored by measuring the absorption spectra of the solution at 10 min intervals to determine the dye concentration. The MV solution exhibited absorption bands at 582 nm and 334 nm, respectively, and the absorbance decreased over time, indicating the decay of the MV (Figure 8a). Moreover, the color of the solution became fainter as the dye was progressively degraded. The observed removal rate of the MV was 88.93% after 120 min.

For comparison, we also studied the degradation of NPh under the same conditions (see Figure S1 in Supplementary Information), which was 81.37% after 120 min.

### 3.8. Kinetic Studies

**Figure 9:**
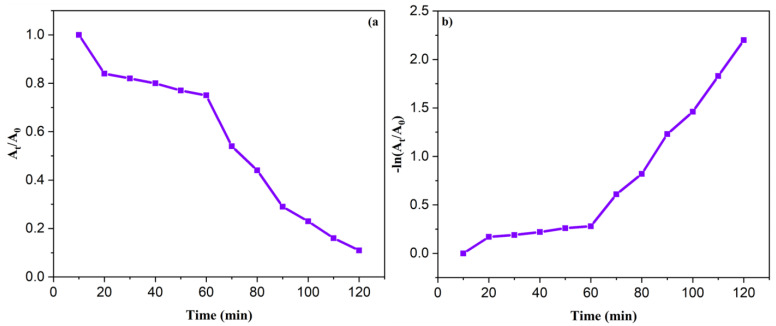
Variation of *A_t_*/*A*_0_ (**a**) and ln (*A_t_*/*A*_0_) (**b**) with time for Ag/ZnO NPs in the degradation of MV.

The photocatalytic degradation of MV by the Ag/ZnO NPs was analyzed using the pseudo-first-order kinetics model, which is described by the following formula:

$$\ln\frac{A_{0}}{A_{t}}=k\;t$$

where *A*_0_ represents the initial absorbance of the dye at time zero, *A_t_* is the absorbance of the dye at a given time (*t*), and *k* is the rate constant. The graph of *A_t_*/*A*_0_ versus the time was plotted and an upward slope confirmed that the pseudo-first-order model was a good fit for the degradation of the MV, as shown in Figure 9 and Table 1. The coefficient of determination (R^2^) for the MV was found to be 0.88766, which was smaller compared to the value of the NPh (0.98003; Figure S2 and Table S1, Supplementary Information), which also followed a pseudo-first-order model.

### 3.9. Factors Affecting % Removal of MV

#### 3.9.1. Effect of Catalyst Concentration

Different amounts of catalysts (3, 5, 7, and 10 mg) were used to investigate the effect of the catalyst concentration on the degradation rate, while keeping the reaction conditions constant. The results showed that the degradation rate augmented as the catalyst amount increased from 3 to 5 mg, and then decreased as the amount of catalyst increased from 7 to 10 mg. The best results were obtained with 5 mg of catalyst in a 10 ppm aqueous solution of MV within 60 min. This can be attributed to the decreased penetration of light intensity, which resulted in more active sites being available for the reaction. As some portion of the catalyst became available, there was less degradation of the dye molecules.

#### 3.9.2. Effect of MV Concentration

**Figure 10:**
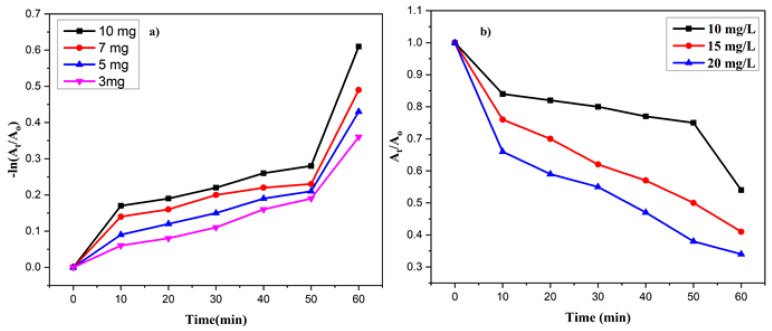
Variation of *A_t_*/*A*_0_ (**a**) and ln (*A_t_*/*A*_0_) (**b**) with time for Ag/ZnO NPs, in the degradation of MV, using different dye concentrations.

To determine the extent of this degradation, various concentrations of MV (10 ppm, 15 ppm, and 20 ppm) were employed (Figure 10).

It was observed that the use of 10 ppm of MV dye resulted in a 45% removal rate. However, as the dye concentration increased, the degradation rate decreased. The degradation of pollutants is dependent on the production of OH radicals and holes, which then react with the organic pollutant. With an increase in the initial concentration of the MV, the possibility of a reaction between dyes and radicals rises. Therefore, as the dye concentration increased, the removal rate decreased. This was due to the reduced production of radicals on the catalyst surface at high MV concentrations. Additionally, aggregation with the catalyst may have occurred due to an increase in the initial amount of MV. The zone where high light intensity is irradiated is where a higher degradation of pollutants occurs. However, the formation of radicals and the absorption of visible light by the dye, instead of a catalyst, may have decreased the efficiency of the reaction, resulting in a reduced degradation rate.

#### 3.9.3. Effect of pH

**Figure 11:**
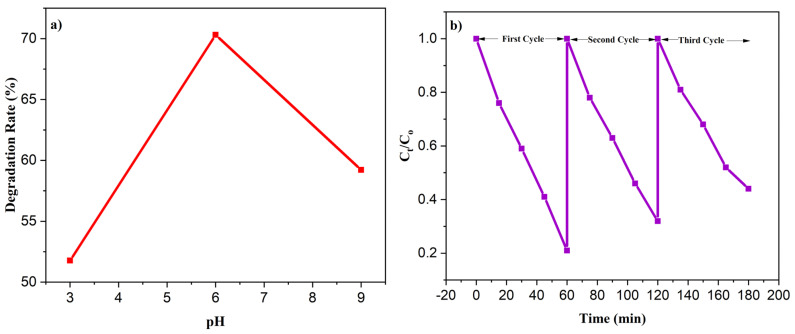
(**a**) Effect of pH on degradation rate of MV, (**b**) reusability of photocatalyst.

The degradation of the MV dye was carefully monitored under different pH values (pH 3, 6, and 9), using a dye concentration of 10 ppm, as depicted in Figure 11a. To achieve the desired acidic and basic pH levels, concentrated HCl and NaOH were added in varying concentrations to the dye solution. The obtained results reveal a notable removal rate at acidic pH levels, which gradually decreased as the pH became more alkaline.

Specifically, our findings demonstrate that, at a pH of 6, approximately 70.31% of the MV dye was destabilized within 1 h. However, when the pH was raised to 9, the removal rate dropped to 59.22%. This behavior can be attributed to the strong electrostatic interactions between the MV dyes and the surface of the photocatalyst. At a pH of 6, the degradation efficiency was enhanced due to an increased charge density, resulting in improved electrostatic interactions between the dye and the photocatalyst.

Conversely, as the pH increased to 9, the charge density of the MV decreased, leading to a reduction in the degradation efficiency. Additionally, repulsive forces began to act between the photocatalyst and the dye molecules at higher pH levels, further decreasing the degradation rate. Furthermore, the decrease in OH radicals, which are potent oxidizing agents responsible for oxidizing the MV dye molecules, also contributed to the decline in the removal rate at higher pHs.

#### 3.9.4. Reusability of Photocatalyst

To assess the recycling capability of the prepared photocatalyst, it was subjected to washing with deionized water and ethanol, followed by drying. The reusability tests were conducted for a total of three cycles and the results are presented in Figure 11b.

Upon analyzing the data, it was observed that the photocatalytic performance of the MV degradation gradually decreased from an initial efficiency of 88.41% to 76.92% after three cycles. This notable decline in performance demonstrated the remarkable reusability and stability of the photocatalyst [44,45].

The decrease in the degradation effectiveness can be attributed to the adsorption of intermediate compounds onto the surface of the photocatalyst. As the photocatalytic reaction proceeded, these intermediate compounds tended to accumulate on the photocatalyst surface over successive cycles. This accumulation hindered the active sites on the photocatalyst from efficiently interacting with the MV dye molecules, thereby reducing the degradation efficiency.

### 3.10. Mechanism of Photocatalytic Degradation

**Figure 12:**
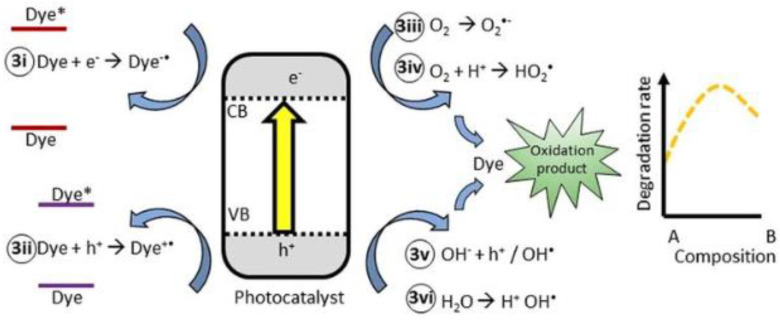
Mechanism for degradation of MV by Ag/ZnO.

Photocatalytic oxidation was utilized to degrade the MV dye [46,47,48]. This method relies on the use of light to initiate a reaction and activate principal species to detoxify organic pollutants. The process is based on electronic transitions, where the band gap energy of the photocatalyst is lower than the absorption energy. When the used solar light energy introduced to an aqueous dye solution containing a catalyst is larger than the band energy, an electron is excited from the valence band (VB) to the conduction band (CB), leaving a hole in its position (h^+^_VB_). The reduction of molecular oxygen into superoxide radical anion (O_2_˙) occurs via a reaction with the electron. The valence band hole transforms hydroxyl ions or water molecules into (OH˙). The electron-hole pair has a strong oxidizing ability and, along with radicals, leads to the degradation of organic dye (as shown in Figure 12). The possible reactions involved in this experiment are [49]:

Ag/ZnO NPs + visible light → e^−^_CB_ + h^+^_VB_

e^−^_CB_ + O_2_ → O_2_

h^+^_VB_ + H_2_O/OH → OH

OH + MV → degradation

e^−^_CB_ + pollutants → reduction product

h^+^_VB_ + pollutants → oxidation product

### 3.11. Comparison with Literature

Table 2 lists several studies on the degradation of MV. It can be seen that Sathish et al. achieved the maximum degradation of MV in the shortest time [50], but they used UV light and NaBH_4_. Compared to other authors who also used Ag/ZnO composites [51,52,53], our results were better, as we achieved 88.9% in 120 min using sunlight. Subhan et al. [51] achieved a slightly better result (93%), but they needed more time (210 min) and used a mercury lamp. Hosseini et al. [52] achieved 97%, but they needed 180 min and used a UV lamp. Kumaresan et al. [53] only achieved 77.6% in 100 min and also used a UV lamp. Other researchers reported more than 90% degradation using a catalyst prepared via the hydrothermal method [54,55,56,57], but this approach suffers from low yields and high raw material inputs. In order to overcome these limitations, we developed a high-yield, eco-friendly synthesis method for Ag/ZnO using *Leptadenia pyrotechnica* leaf extract. This approach not only avoids the use of synthetic catalysts, but also achieves high degradation percentages in a shorter amount of time. Furthermore, this study represents the first use of this plant extract for the synthesis of metallic nanoparticles, demonstrating its potential as a green synthesis route for future research.

A comparison of several studies dealing with the degradation of NPh is given in Table S2 of Supplementary Information.

## 4. Conclusions

The synthesis of NPs using plant extracts shows several advantages, such as a non-toxic and environmentally friendly nature, compared to chemical methods. Bimetallic NPs of Ag and ZnO were synthesized using *Leptadenia pyrotechnica* leaf extract for the first time. The results of this study indicate that Ag/ZnO bimetallic NPs are more effective than monometallic Ag and ZnO NPs, exhibiting strong antioxidant activity at different concentrations. Furthermore, these BNPs demonstrated the highest values for biological activities, such as antibacterial activity, antioxidant activity, and cytotoxicity, as well as excellent photocatalytic degradation of MV and NPh. The antibacterial activity results show that the bimetallic NPs inhibited the growth of both Gram-positive (*S. aureus*) and Gram-negative (*E. coli*) bacteria. The cytotoxicity results indicate that a controlled quantity of monometallic and bimetallic NPs had no adverse effects on cell viability. However, the observed decrease in cell (L-929) viability suggests that monometallic and bimetallic NPs may have anticancer properties. Further comprehensive studies are necessary to assess their pharmacological properties and toxicity. The degradation rates of MV and NPh monitored over 120 min were 88.93% and 81.37, respectively. The degradation kinetics for MV and NPh followed a pseudo-first-order model.

## Figures and Tables

**Table 1:** Statistical analysis of Ag/ZnO NPs for MV degradation over time.

Time (min)	Position	Model	Residual	Residual Square	RMSE	R^2^	Standard Deviation
10	0.17	1.068	−0.898	0.806404	3.3387	0.88766	0.286065
20	0.19	1.966	−1.776	3.154176	3.3185		
30	0.22	2.864	−2.644	6.990736	3.2383		
40	0.26	3.762	−3.502	12.264004	3.0531		
50	0.28	4.66	−4.38	19.1844	2.6977		
60	0.61	5.558	−4.948	24.482704	2.0530		
70	0.64	6.456	−5.816	33.825856	8.0551		
80	0.69	7.354	−6.664	44.408896	7.6972		
90	0.73	8.252	−7.522	56.580484	7.2004		
100	0.81	9.15	−8.34	69.5556	6.5128		
110	0.86	10.048	−9.188	84.419344	5.5519		
120	0.92	10.946	−10.026	100.520676	4.0931		

**Table 2:** Degradation of MV by several different photocatalysts.

Photocatalyst	Synthesis Method	Time for Degradation (min)	Light Source	Degradation Rate (%)	Reference
Ag/ZnO Bimetallic Nanoparticle	Hydrothermal	30	LED lamp	98.6	[55]
TiSiW_12_O_40_/TiO_2_	Crystallization	180	Xenon lamp	93.2	[58]
Fe_2_O_3_ Nanoparticles	Hydrothermal	120	Visible light	87.6	[59]
ZnO Nanoparticles	Supercritical antisolvent precipitation	60	Visible light	95.4	[60]
Ag Nanoparticles	Chemical reduction	45	Xenon lamp	88.3	[61]
CdS Nanoparticles	Hydrothermal	50	Visible light	96.7	[54]
Co_3_O_4_ Nanoparticles	Co-precipitation	180	UV lamp	82.4	[62]
CuO Nanoparticles	Hydrothermal	90	Visible light	94.5	[56]
Ag/ZnO nanoparticles	Co-precipitation	210	Mercury lamp	93	[51]
Ag doped ZnO nanoparticles	Microwave irradiation method	100	UV lamp	77.6	[53]
Ag/ZnO nanocomposites	Co-precipitation	8	UV lamp	97	[50]
Ag doped ZnO nanoparticles	Precipitation	180	UV lamp	95	[52]
ZnO/CNA material	Impregnation method	120	Sunlight	49.7	[63]
SnO_2_ Nanoparticle	Hydrothermal	120	Sunlight	80	[64]
TiO_2_ Nanocomposites	Hydrothermal	180	Sunlight	98	[57]
Ag/ZnO nanoparticles	Co-precipitation	120	Sunlight	88.9	Our work

## Data Availability

Data will be made available upon request.

## Supplementary Materials

The following supporting information can be downloaded at: https://www.mdpi.com/article/10.3390/nano13142079/s1, Figure S1. (a) Photocatalytic degradation and (b) degradation rate (%) of NPh on Ag/ZnO NPs. Figure S2. Variation of At/A0 (a) and ln (At/A0) (b) with time for Ag/ZnO NPs in the degradation of NPh. Table S1. Statistical analysis of Ag/ZnO NPs for NPh degradation over time. Table S2. Degradation of NPh by several different photocatalysts. References [65,66,67,68,69,70,71,72,73,74,75,76,77,78,79] are cited in the supplementary materials.
