# Supplementary material for: The Biogenic Synthesis of Bimetallic Ag/ZnO Nanoparticles: A Multifunctional Approach for Methyl Violet Photocatalytic Degradation and the Assessment of Antibacterial, Antioxidant, and Cytotoxicity Properties
Source: Nanomaterials (Basel). 2023 Jul 15;13(14):2079. doi: 10.3390/nano13142079 (PMC10385465; doi:10.3390/nano13142079)
Supplement: Supplementary file 1 [file nanomaterials-13-02079-s001.zip › nanomaterials-2461234-supplementary.pdf]

Supplementary Materials

The Biogenic Synthesis of Bimetallic Ag/ZnO Nanoparticles: A Multifunctional Approach for Methyl Violet Photocatalytic Degradation and the Assessment of Antibacterial, Antioxidant, and Cytotoxicity Properties

Figures

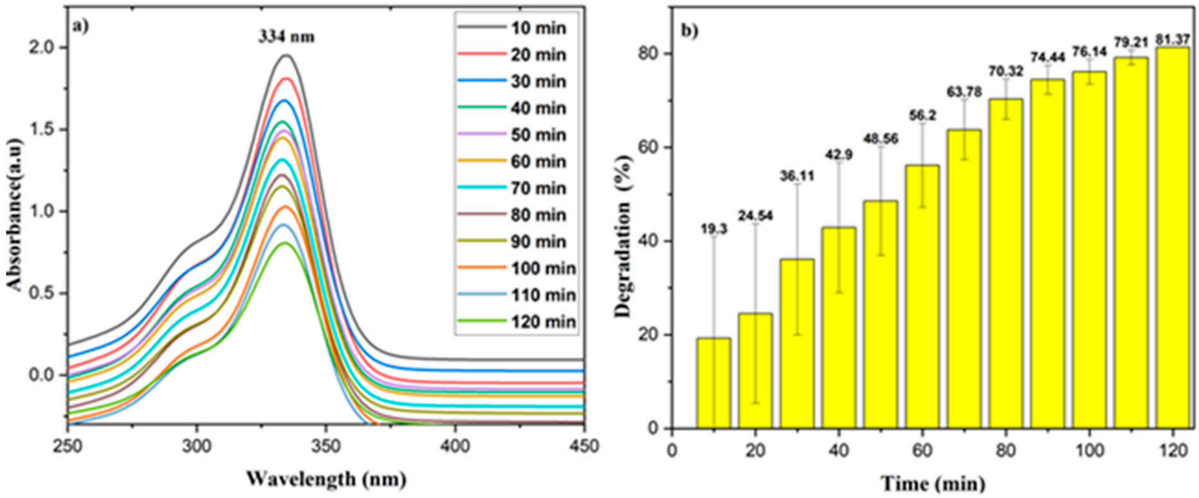

Figure S1. (a) Photocatalytic degradation and (b) degradation rate (%) of NPh on Ag/ZnO NPs.

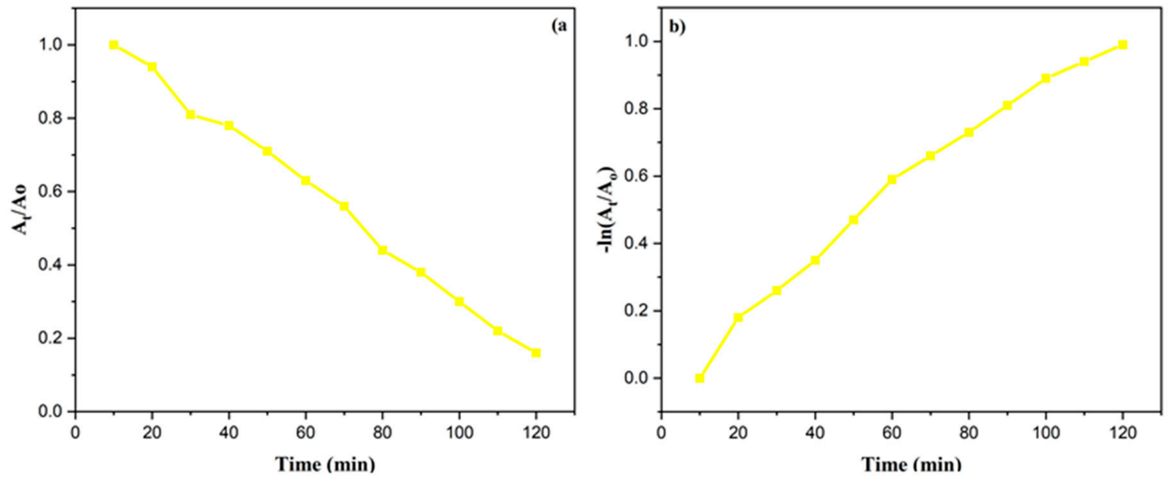

Figure S2. Variation of A_t/A_0 (a) and ln (A_t/A_0) (b) with time for Ag/ZnO NPs in the degradation of NPh.

Tables

Table S1. Statistical analysis of Ag/ZnO NPs for NPh degradation over time.

Time (min)	Position	Model	Residual	Residual square	RMSE	R^2
10	0.15	1.048	-1.856	3.444736	6.6738	
20	0.21	2.006	-2.774	7.695076	6.6523	
30	0.29	2.984	-3.642	13.264164	6.6039	0.98003
40	0.34	3.932	-4.53	20.5209	6.5197	
50	0.38	4.87	-5.428	29.463184	6.3872	
60	0.42	5.808	-6.416	41.165056	6.1920	
70	0.55	6.836	-7.304	53.348416	5.9085	
80	0.67	7.854	-8.152	66.455104	5.5195	
90	0.74	8.822	-9.07	82.2649	4.9927	
100	0.83	9.81	-9.928	98.565184	4.2511	
110	0.88	10.758	-10.836	117.418896	3.1398	
120	0.94	11.716	0.94	0.8836	0.2713	

Table S2. Degradation of NPh by several different photocatalysts.

Photocatalyst	Synthesis Method	Time for Degradation (min)	Light Source	Degradation Rate (%)	Reference
**ZnO/CdO**	Pulsed laser deposition	200	UV light	98.3	[1]
**CuO@SiO$_2$**	Reflux	12	Visible light	100	[2]
**Ag Nanoparticles**	Pulsed laser deposition	60	UV light	80	[3]
**Au/rGO**	Spray drying method	30	Visible light	95.1	[4]
**Ag/Almond Shell**	Green Synthesis	4.5	Visible Light	100	[5]
**Ag doped Fe$_3$O$_4$/C**	Solvothermal	26	Visible light	100	[6]
**Au/TiO$_2$**	Photoreduction deposition	6	Visible light with excess NaBH$_4$	100	[7]
**PVA/Ag**	Pulsed Laser Ablation	25	Visible light with excess NaBH$_4$	93	[8]
**Ag doped CuO**	Pulsed laser deposition	25	Visible light with excess NaBH$_4$	100	[9]
**Ag nanoparticles**	Green Synthesis	53	Visible light with excess NaBH$_4$	100	[10]
**CuO**	Green Synthesis	30	Visible light with excess NaBH$_4$	87	[11]
**CuO/clinoptilolite**	Green Synthesis	150 sec	Visible light with excess NaBH$_4$	100	[12]
**ZnO**	Microwave irradiation method	180	UV light	100	[13]
**MOF[Zn(BDC)DMF]**	Ultrasound irradiation and solvothermal	10	Sunlight	100	[14]
**Ag/MWCNTs**	Biological Method	50	Visible light with excess NaBH$_4$	94	[15]
**Ag/ZnO nanoparticles**	Co-precipitation	120	Sunlight	81.37	Our work

References

[1] Mostafa, A. M., & Mwafy, E. A. (2020). Synthesis of ZnO/CdO thin film for catalytic degradation of 4-nitrophenol. *Journal of Molecular Structure*, *1221*, 128872.

[2] Ramya, E., Thirumurugan, A., Rapheal, V. S., & Anand, K. (2019). CuO@ SiO2 nanoparticles assisted photocatalytic degradation of 4-nitrophenol and their antimicrobial activity studies. *Environmental Nanotechnology, Monitoring & Management*, *12*, 100240.

[3] Zakaria, M. A., Menazea, A. A., Mostafa, A. M., & Al-Ashkar, E. A. (2020). Ultra-thin silver nanoparticles film prepared via pulsed laser deposition: synthesis, characterization, and its catalytic activity on reduction of 4-nitrophenol. *Surfaces and Interfaces*, *19*, 100438.

[4] Li, N., Zhang, F., Wang, H., & Hou, S. (2019). Catalytic degradation of 4-nitrophenol in polluted water by three-dimensional gold nanoparticles/reduced graphene oxide microspheres. *Engineered Science*, *7*(4), 72-79.

[5] Bordbar, M. (2017). Biosynthesis of Ag/almond shell nanocomposite as a cost-effective and efficient catalyst for degradation of 4-nitrophenol and organic dyes. *RSC advances*, *7*(1), 180-189.

[6] Chishti, A. N., Guo, F., Aftab, A., Ma, Z., Liu, Y., Chen, M., ... & Diao, G. (2021). Synthesis of silver doped Fe3O4/C nanoparticles and its catalytic activities for the degradation and reduction of methylene blue and 4-nitrophenol. *Applied Surface Science*, *546*, 149070.

[7] Ren, Z. H., Li, H. T., Gao, Q., Wang, H., Han, B., Xia, K. S., & Zhou, C. G. (2017). Au nanoparticles embedded on urchin-like TiO2 nanosphere: an efficient catalyst for dyes degradation and 4-nitrophenol reduction. *Materials & Design*, *121*, 167-175.

[8] Mostafa, A. M., & Menazea, A. A. (2020). Polyvinyl Alcohol/Silver nanoparticles film prepared via pulsed laser ablation: An eco-friendly nano-catalyst for 4-nitrophenol degradation. *Journal of Molecular Structure*, *1212*, 128125.

[9] Menazea, A. A., & Mostafa, A. M. (2020). Ag doped CuO thin film prepared via pulsed laser deposition for 4-nitrophenol degradation. *Journal of Environmental Chemical Engineering*, *8*(5), 104104.

[10] Bogireddy, N. K. R., Pal, U., Gomez, L. M., & Agarwal, V. (2018). Size controlled green synthesis of gold nanoparticles using Coffea arabica seed extract and their catalytic performance in 4-nitrophenol reduction. *RSC advances*, *8*(44), 24819-24826.

[11] Singh, S., Kumar, N., Kumar, M., Agarwal, A., & Mizaikoff, B. (2017). Electrochemical sensing and remediation of 4-nitrophenol using bio-synthesized copper oxide nanoparticles. *Chemical engineering journal*, *313*, 283-292.

[12] Bordbar, M., Sharifi-Zarchi, Z., & Khodadadi, B. (2017). Green synthesis of copper oxide nanoparticles/clinoptilolite using Rheum palmatum L. root extract: high catalytic activity for reduction of 4-nitro phenol, rhodamine B, and methylene blue. *Journal of sol-Gel science and Technology*, *81*, 724-733.

[13] Qin, Y., Zhang, H., Tong, Z., Song, Z., & Chen, N. (2017). A facile synthesis of Fe3O4@ SiO2@ ZnO with superior photocatalytic performance of 4-nitrophenol. *Journal of environmental chemical engineering*, *5*(3), 2207-2213.

[14] Samuel, M. S., Bhattacharya, J., Parthiban, C., Viswanathan, G., & Singh, N. P. (2018). Ultrasound-assisted synthesis of metal organic framework for the photocatalytic reduction of 4-nitrophenol under direct sunlight. *Ultrasonics sonochemistry*, *49*, 215-221.

[15] Song, X., & Shi, X. (2017). Bioreductive deposition of highly dispersed Ag nanoparticles on carbon nanotubes with enhanced catalytic degradation for 4-nitrophenol assisted by Shewanella oneidensis MR-1. *Environmental Science and Pollution Research*, *24*, 3038-3044.
